# Physical Activity and Bone Mineral Accrual in Boys with Different Body Mass Parameters during Puberty: A Longitudinal Study

**DOI:** 10.1371/journal.pone.0107759

**Published:** 2014-10-03

**Authors:** Donvina Vaitkeviciute, Evelin Lätt, Jarek Mäestu, Toivo Jürimäe, Meeli Saar, Priit Purge, Katre Maasalu, Jaak Jürimäe

**Affiliations:** 1 Institute of Sport Pedagogy and Coaching Sciences, Faculty of Exercise and Sport Sciences, Centre of Behavioral, Social and Health Sciences, University of Tartu, Tartu, Estonia; 2 Department of Traumatology and Orthopedics, Faculty of Medicine, University of Tartu, Tartu, Estonia; Harvard Medical School, United States of America

## Abstract

The aim of our longitudinal study was to investigate the relationships between physical activity and bone mass in boys with different body mass status during the years surrounding pubertal growth spurt. Two hundred and six boys entering puberty took part in this study. The subjects were divided into underweight (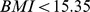), normal weight (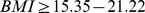), overweight (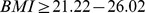) and obese (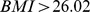) groups at baseline according to age related categories. Whole-body DXA scans were performed at baseline, after 12 and 24 months to assess body composition (lean body mass, fat mass), and total body (TB), lumbar spine (LS) and femoral neck (FN) bone mineral density (BMD) parameters. Physical activity was measured by 7- day accelerometry. For longitudinal analysis, multilevel fixed effects regression models were constructed. Biological age, height and lean body mass had an effect for explanation of TB BMD, FN BMD and LS BMD. Moderate to vigorous physical activity (MVPA), vigorous physical activity (VPA) and sedentary time (SED) had the significant effect only on FN BMD. Being an underweight boy at the baseline indicated greater chance (p<0.01) to have lower TB BMD in the future (2 years at follow up) development, compared to normal weight (estimates = −0.038), overweight (estimates = −0.061) and obese boys (estimates = −0.106).

## Introduction

Osteoporosis is the most common metabolic skeletal disease in humans and is a major public health problem. Optimising peak bone mass during puberty is an important key factor for the prevention of osteoporosis in later years [1]. Pubertal growth spurt is a period of rapid acceleration in the growth velocity of almost all skeletal tissue. Data from the longitudinal studies of bone mineral accrual reveal that the maximum bone growth velocity occurs during the 4-year period surrounding peak height velocity (PHV) with approximately 26% of adult total body bone mineral gained [2], [3], [4]. Peak height velocity (PHV) is an important somatic biological maturity parameter, widely used in growth studies and allows subjects to be compared by biological rather than by chronological age [5]. Underweight boys have been characterized by lower bone mineral density (BMD) compared to their normal weight peers and are at an increased risk of fractures [6]. Usually studies have examined differences in BMD between underweight boys attributed to diseases such as anorexia nervosa and their normal weight peers [7], [8]. However, to our best of knowledge, there are no longitudinal studies performed that have investigated bone mineral accrual during puberty in healthy underweight boys.

There is a controversy in the literature as to whether obese boys have higher or lower bone mass compared with normal weights [9], [10]. Studies in the literature are contradictory indicating that obese boys have increased [11], [12], decreased [13], [14], or similar [15], [16] BMD values compared to normal weight peers. However, obese boys are also linked to an increased risk for bone fractures during rapid skeletal growth in pubertal period [17].

Physical activity (PA) is a major environmental factor that positively influences bone mineral accrual during puberty [18], however most studies have been carried out with exercise intervention programs [19]–[22]. Some studies have found positive cross-sectional data [23]–[26] and only one recent longitudinal study [27] investigating bone mineral parameters and PA relationships. However, it is relevant to continue investigating the amount and intensity of PA that could have valuable effect on bone mineral accrual during puberty in boys. The aim of the present study was to investigate the longitudinal relationships between physical activity and bone mineral parameters in boys with different body mass status during pubertal growth spurt.

## Materials and Methods

### Participants and Study Design

Study participants were recruited from different schools in Tartu city (Estonia) and its surrounding areas. At the baseline, the subjects were 12 years old and the measurements continued every year (in total 3 measurement sessions: at baseline, after 12 and 24 months). The present study sample consisted of 206 peripubertal boys. The inclusion criteria for current study were that a boy had to be healthy, took part in obligatory physical education (PE) lessons at school, and had the full set of PA data from all the measurement sessions.

### Ethics statement

The subjects and their parents received information about the study and the procedures. The written signed informed consent was obtained from parents, while children gave the verbal assent. All procedures were reviewed and approved by the Medical Ethics Committee of the University of Tartu, Tartu, Estonia.

### Anthropometry

Height (cm) was measured with a Martin metal anthropometer to the nearest 0.1 cm according to the standard technique (GPM anthropological instruments, Zurich, Switzerland). Body mass (kg) was measured using a medical electronic scale (A&D Instruments, Abingdon, UK) and recorded with 0.05 kg precision with the subject wearing light clothes. Body mass index (BMI; 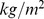) was calculated as body mass (in kilograms) divided by height in square meters. The subjects were divided into underweight (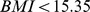), normal weight (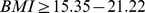), overweight (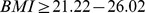) and obese (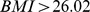) groups at baseline. The cut-off points for BMI were set according to Cole et al. [28].

### Maturity Assessment

Pubertal development was assessed by self-report questionnaire of pubertal stages according to Tanner [29]. Each boy was given line drawings, pictures and descriptions representing genitalia and pubic hair development stages. The subject had to choose the one that most closely matched his own development. Age at peak height velocity (APHV) was assessed using gender specific anthropometric equations according to Mirwald et al. [30]. Biological age was calculated as the years from APHV.

### Bone Densitometry and Body Composition

Bone mineral density (BMD; 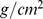) of total body (TB), lumbar spine (LS) and femoral neck (FN), and body composition: fat mass (FM) and lean body mass (LBM) were measured using dual-energy x-ray absorptiometry (DXA; DPX-IQ densitometer, Lunar Corporation, Madison, USA) equipped with proprietary software (version 3.6). Boys were scanned in a supine position wearing light clothing. The medium scan mode and the standard subject positioning was used for total body measurements, which were analyzed using the extended analysis option. To reduce the impact of the operator variability factor, one qualified observer analyzed all scans over the 2-year period. The precision of measurement expressed as coefficient of variation (CV) was less than 2% for all bone mineral and body composition measurements. The ratio of LBM and height (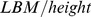) was calculated.

### Physical activity

A uniaxial accelerometer GT1M (ActiGraph, Pensacola, USA) was used to assess physical activity. GT1M accelerometer is a small (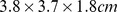) and lightweight (27 g) device that detects vertical accelerations ranging in magnitude from 0.05 to 2.00 G's with a frequency response of 0.25–2.50 Hz. The ActiGraph accelerometer has been previously validated in laboratory and free-living conditions in children and adolescents [31]. All participants wore the accelerometer on the right hip attached by an elastic belt and adjustable buckle for 7 consecutive days. Boys were instructed to remove the devices during showering, bathing, swimming and during sleep period. The used interval of time (epoch) was set at one minute. Data were uploaded to a computer after the measurements and were analyzed later. At least two workdays and one weekend day of recording with minimum 8 hours/day was set as an inclusion criterion and all sequences of 10 min or more of consecutive epoch with 0 counts were removed from the analyzes [32], [33]. Total daily physical activity (TPA;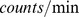) was calculated as the total number of counts divided by total daily registered time. The following PA thresholds were used: sedentary time (SED;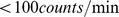), light intensity PA (LPA; 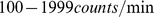), moderate intensity PA (MPA; 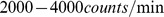) and vigorous intensity PA (VPA; 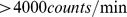) [34]. The time spent in moderate and vigorous intensity PA (MVPA; 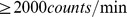) was calculated as the sum of MPA and VPA.

### Statistical Analysis

Statistical analysis was performed using SPSS software version 21.0 (SPSS Inc.) and SAS 9.2 (SAS Institute, Inc. Cary, NC, USA). Descriptive statistics were performed for all variables by BMI group at baseline and were presented as means and standard deviations (± SD). Normality of parameters was controlled by Shapiro-Wilks test and q-q plots. Analysis of variance (ANOVA) with Tukey *post hoc* was used to determine differences between underweight, normal weight, overweight and obese groups at baseline for anthropometric, body composition, maturity, bone mineral and physical activity parameters.

For longitudinal analyses, multilevel fixed effects regression models were constructed using PROC MIXED method (SAS version 9.2). Multilevel modeling allowed us to include participants who randomly missed some of the measurements. These models included time of measurements (0, 12, 24 months), biological age, height, LBM, SED, MVPA and group (underweight, normal weight, overweight and obese at baseline). To allow for the non-linearity biological age function was included into the linear models. The coefficients of fixed variables were used to predict TB BMD, FN BMD and LS BMD. A P-value less than 0.05 was considered significant for all analyses.

## Results

### Descriptive Characteristics

Data on participants' age, maturity, anthropometry, bone mineral parameters and physical activity levels at baseline are presented in Table 1. There was no difference in chronological age among all four groups with different body mass parameters (range, 11.7–12.1 yrs). However, biological age in underweight boys was significantly lower compared to other subgroups (P<0.05). Underweight boys were significantly shorter compared to obese boys. All groups differed significantly from each other by body mass, FM and LBM (P<0.05). In addition, TB BMD indicated significant differences in all four groups at baseline. LS BMD was significantly lower in underweight and normal weigh boys when compared to obese boys (P<0.05), while FN BMD was significantly lower in underweight boys compared to normal weight and obese boys. No differences were found in SED time among all groups of boys. However, underweight and normal weight boys showed higher MVPA and VPA levels compared to obese boys (P<0.05).

**Table 1:** Mean (± SD) characteristics of the subjects at baseline measurement.

	Underweight	Normal weight	Overweight	Obese
	(n = 27)	(n = 133)	(n = 22)	(n = 24)
**Age (years)**	11.7±0.47	12.1±0.69	11.9±0.74	12.1±0.95
**Biological age**	−2.03±0.48^b^ ^c^ ^d^	−1.50±0.73^a^ ^d^	−1.39±0.54^a^ ^d^	−0.66±0.78^a^ ^b^ ^c^
**Tanner stage**	2.29±0.47^b^	2.81±0.72^a^	2.41±0.67	2.79±0.78
I/II/III/IV/V	0/19/8/0/0	5/34/75/19/0	1/12/8/1/0	1/7/12/4/0
**Height (cm)**	152.0±6.86^d^	153.9±8.62^d^	154.6±7.06^d^	160.8±7.46^a^ ^b^ ^c^
**Body mass (kg)**	35.2±3.98^b^ ^c^ ^d^	43.6±6.74^a^ ^c^ ^d^	56.9±5.43^a^ ^b^ ^d^	75.3±11.02^a^ ^b^ ^c^
**BMI (g/cm^2^)**	15.1±0.59^b^ ^c^ ^d^	18.3±1.44^a^ ^c^ ^d^	23.7±1.06^a^ ^b^ ^d^	28.9±2.23^a^ ^b^ ^c^
**FM (kg)**	4.4±1.17^b^ ^c^ ^d^	7.9±2.78^a^ ^c^ ^d^	18.6±4.28^a^ ^b^ ^d^	30.9±6.54^a^ ^b^ ^c^
**LBM (kg)**	28.7±3.52^b^ ^c^ ^d^	33.3±5.97^a^ ^d^	34.5±5.08^a^ ^d^	40.8±6.36^a^ ^b^ ^c^
**LBM/height**	0.18±0.143^b^ ^c^ ^d^	0.21±0.212^a^ ^c^ ^d^	0.23±0.271^a^ ^b^ ^d^	0.25±0.289^a^ ^b^ ^c^
**TB BMD (g/cm^2^)**	0.932±0.053^b^ ^c^ ^d^	0.979±0.057^a^ ^d^	1.001±0.047^a^ ^d^	1.056±0.067^a^ ^b^ ^c^
**LS BMD (g/cm^2^)**	0.796±0.088^d^	0.829±0.098^d^	0.849±0.094	0.898±0.084^a^ ^b^
**FN BMD (g/cm^2^)**	0.852±0.077^b^ ^d^	0.906±0.099^a^	0.913±0.092	0.938±0.088^a^
**SED (min/day)**	416.0±54.2	410.5±65.5	406.1±72.9	411.7±62.9
**MVPA (min/day)**	64.1±34.8^d^	60.6±25.8^d^	50.6±15.2	40.9±25.8^a^ ^b^
**VPA (min/day)**	15.3±16.1^d^	12.8±11.7^d^	7.2±4.6	5.1±7.7^a^ ^b^

a Significantly different from underweight boys;

b significantly different from normal weight boys;

c significantly different from overweight boys;

d significantly different from obese boys (P<0.05);

Biological age is years from APHV (age at peak height velocity); BMI, body mass index; TB BMD, total body bone mineral density; FN BMD, femoral neck bone mineral density; LS BMD, lumbar spine bone mineral density; SED, sedentary time; MVPA, moderate and vigorous physical activity.

There was an increase in SED time for all the age groups during the 24-month study period (Table 2). Further, a significant decrease was seen in light PA at all measurement points. No change was seen in MPA, except for underweights where MPA had decreased at 24-month follow-up compared to baseline. Vigorous PA increased significantly for all weight groups (except for underweights) at 24 months follow-up compared to baseline.

**Table 2:** The change of PA levels during the study period in four different groups of participants.

	At baseline	After 12 months	After 24 months
	Underweight		
	(n = 24)	(n = 22)	(n = 22)
**SED (min/day)**	416.0±54.2	463.7±72.2^1^	565.7±111.3^1^ ^2^
**LPA (min/day)**	305.7±54.3	271.7±43.4^1^	173.5±48.4^1^ ^2^
**MPA (min/day)**	48.8±21.9	42.2±16.3	34.7±16.9^1^
**VPA (min/day)**	15.3±16.1	18.2±14.7	22.2±16.8
**MVPA (min/day)**	64.1±34.8	60.5±28.7	56.9±32.2

	Normal weight		
	(n = 110)	(n = 92)	(n = 90)
**SED (min/day)**	410.5±65.5	424.3±69.1	583±91.7^1^ ^2^
**LPA (min/day)**	308.2±59.3	278.2±53.9^1^	174.3±43.1^1^ ^2^
**MPA (min/day)**	47.9±18.5	43.1±17.4	36.3±13.5^1^
**VPA (min/day)**	12.8±11.7	15.9±13.6	20.6±16.2^1^
**MVPA (min/day)**	60.6±25.8	59.0±26.9	56.9±25.3

	Overweight		
	(n = 21)	(n = 17)	(n = 18)
**SED (min/day)**	406.1±72.9	433.3±51.1	582.1±73.8^1^ ^2^
**LPA (min/day)**	315.6±54.5	270.0±65.3	182.5±43.8^1^ ^2^
**MPA (min/day)**	43.7±14.1	40.9±24.3	37.0±13.7
**VPA (min/day)**	7.2±4.6	12.0±7.9	15.2±10.1^1^
**MVPA (min/day)**	50.6±15.2	52.9±27.4	52.2±18.4

	Obese		
	(n = 23)	(n = 20)	(n = 17)
**SED (min/day)**	411.7±62.9	431.4±66.3	565.7±74.7^1^ ^2^
**LPA (min/day)**	312.9±66.3	263.6±62.1	165.5±44.9^1^ ^2^
**MPA (min/day)**	35.5±19.9^b^	34.7±17.6	38.9±14.8
**VPA (min/day)**	5.1±7.7^a^ ^b^	9.5±7.9	14.3±10.2^1^
**MVPA (min/day)**	40.9±25.8^a^ ^b^	44.2±22.4	53.2±23.1

a Significantly different from underweight boys;

b significantly different from normal weight boys; (P<0.05);

1 significantly different from baseline PA level;

2 significantly different from PA level after 12 month;

SED, sedentary time; LPA, light physical activity; MPA, moderate physical activity; VPA, vigorous physical activity; MVPA, moderate to vigorous physical activity.

### Multilevel Regression Models for TB, FN and LS BMD

The variance of intercept for all models was significant, indicating that subjects varied significantly at each measurement occasion in their level of TB BMD (P<0.001), FN BMD (P<0.001) and LS BMD (P<0.001) (Tables 3 and 4). Multilevel models indicated that biological age, height and LBM had the significant effect for explanation of TB BMD, FN BMD and LS BMD in pubertal boys with different body mass values. However, the effect of LBM on TB BMD was not seen, but very close of being significant in the model if testing for the effect of SED time and VPA (P = 0.050; Table 4). SED time and MVPA had a significant effect only in the explanation of FN BMD (P = 0.002 and P = 0.006, respectively; Table 3) as well as for SED and VPA (P = 0.001; P<0.001, respectively; Table 4). However, time (testing at baseline, after 12 and after 24 months) had no effect on bone mineral parameters.

**Table 3:** Multilevel regression model for TB BMD, FN BMD and LS BMD controlling for biological age, height, lean body mass and testing for SED and MVPA.

Variables	TB BMD		FN BMD		LS BMD	
*Fixed effect*	Estimates ± SE	P value	Estimates ± SE	*p* value	Estimates ± SE	*P* value
Intercept	2.3717±0.2727	**<0.001**	1.8821±0.5465	**<0.001**	2.9526±0.4983	**<0.001**
Time	0.002705±0.0036	0.459	−0.00057±0.0066	0.931	0.000131±0.0064	0.983
Biological age	−0.01940±0.0034	**<0.001**	−0.01412±0.0068	**0.042**	−0.02969±0.0063	**<0.001**
Biological age^2^	0.000065±0.00001	**<0.001**	0.000048±0.00002	**0.032**	0.000099±0.00002	**<0.001**
Height	0.02135±0.0057	**<0.001**	0.02826±0.0108	**0.010**	0.03115±0.01032	**0.003**
Lean body mass	0.001090±0.0005	**0.049**	0.002926±0.0010	**0.006**	0.004438±0.0010	**<0.001**
SED	−0.00001±0.00001	0.528	−0.00010±0.00003	**0.002**	−0.00001±0.00003	0.871
MVPA	0.000049±0.00006	0.421	0.000331±0.0001	**0.006**	0.000015±0.0001	0.892
Group	0.02520±0.0043	**<0.001**	0.007300±0.0073	0.321	0.001637±0.0071	0.818

SE is standard error. Biological age - years from age at peak height velocity (APHV). SED - sedentary time. MVPA - moderate to vigorous physical activity. Group effect is four different groups according to baseline BMI.

**Table 4:** Multilevel regression model for TB BMD, FN BMD and LS BMD values when controlling for biological age, height, lean mass and testing for SED and VPA.

Variables	TB BMD		FN BMD		LS BMD	
*Fixed effect*	Estimates ± SE	*P* value	Estimates ± SE	*P* value	Estimates ± SE	P value
Intercept	2.3514±0.2715	**<0.001**	1.8034±0.5430	**<0.001**	2.9229±0.4971	**<0.001**
Time	0.002310±0.0036	0.528	−0.00285±0.0066	0.666	−0.00036±0.0064	0.956
Biological age	−0.01912±0.0034	**<0.001**	−0.01289±0.0068	**0.061**	−0.02939±0.0063	**<0.001**
Biological age^2^	0.000064±0.00001	**<0.001**	0.000043±0.00002	**0.049**	0.000098±0.00002	**<0.001**
Height	0.02115±0.0057	**<0.001**	0.02823±0.0107	**0.009**	0.03055±0.01029	**0.003**
Lean body mass	0.001078±0.0005	0.050	0.002920±0.0010	**0.005**	0.004354±0.0010	**<0.001**
SED	−0.00001±0.00002	0.519	−0.00010±0.00003	**<0.001**	−0.00001±0.00003	0.936
VPA	0.000214±0.00013	0.108	0.001033±0.00025	**<0.001**	0.000265±0.0002	0.284
Group	0.02574±0.0043	**<0.001**	0.009473±0.0073	0.196	0.002946±0.0071	0.679

SE is standard error. Biological age - years from age at peak height velocity (APHV). SED - sedentary time. VPA - vigorous physical activity. Group effect is four different groups according to baseline BMI.

The group effect was significant only for TB BMD (estimates = 0.02520; P<0.001).

As four different BMI groups form group effect, we had to run further the mixed procedure (Table 5) to find which groups vary differently from each other. The further mixed procedure indicated that there was no significant difference for longitudinal model effect on TB BMD between normal weight and overweight groups (P = 0.084), but the rest grouping had significant differences for longitudinal model effect on TB BMD.

**Table 5:** Differences between groups on the effect of model to TB BMD.

Effect	Group	Group	Estimates	*P* value
Group	Underweight	Normal weight	−0.03831±0.01187	**<0.001**
Group	Underweight	Overweight	−0.06080±0.01615	**<0.001**
Group	Underweight	Obese	−0.1056±0.01577	**<0.001**
Group	Normal weight	Overweight	−0.02250±0.01294	0.084
Group	Normal weight	Obese	−0.06726±0.01247	**<0.001**
Group	Overweight	Obese	−0.04476±0.01660	**0.008**

**Figure 1:**
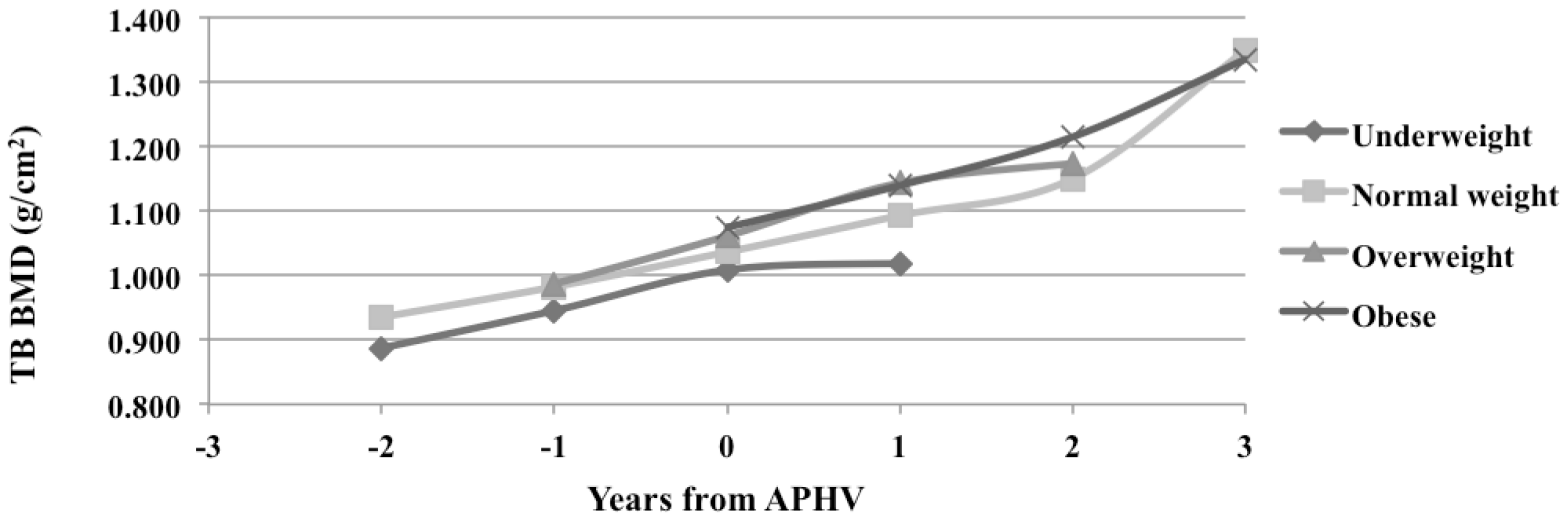
TB BMD changes during pubertal growth spurt. Comparing four different groups – underweight, normal weight, overweight and obese pubertal boys. APHV - age of peak height velocity.

The significant variance of biological age suggests that with the increasing biological age, boys varied by the changes in TB BMD, FN BMD and LS BMD. The tendency of variance could be seen in Figure 1, which presents changes of TB BMD during pubertal growth spurt, where 0 is APHV. We used the third year data to present this variance of changes and it indicated that all obese boys reached their APHV of being approximately 14 years old (variation range of biological age was 0–3 APHV for obese boys). While other groups had greater range of variation, their biological age varied from −2 to 3 APHV.

## Discussion

In this study we aimed to investigate the relationships between PA and bone mass parameters in boys with different body mass values during the pubertal growth spurt. The current findings of multilevel regression models for longitudinal data indicated that MVPA, VPA and SED time had an effect on FN BMD over the 2-year period. However, no effect in the current study was found on TB BMD or LS BMD in boys during the puberty. Our findings confirm the results from the recent cross-sectional study of Kriemler et al. [24], who also found that VPA had significant effect on FN BMD in boys during pubertal growth spurt. Different authors have argued that FN is the site that is most responsive to PA, as loading forces have the most direct impact [35], [36]. Our study results also indicated a decrease in overall physical activity in boys at the beginning of puberty and during further maturation (see Table 2). Such decrease in habitual physical activity and increase in sedentary time with age is well known [37]. Differently from our results, Sherar et al. [38] found the faster decrease in VPA compared to MVPA in children aged 8 to 13 years old. However, in contrast, we found increase of VPA between the time points in every BMI group. This might be due to increase of participation in sports clubs or higher exercise intensities during the physical education lessons.

Underweight boys are less studied compared to underweight girls or overweight and obese boys. According to Misra et al. [8], one of the best predictors of BMD in underweights is LBM. In the current study, underweight boys had significantly lower absolute LBM values compared with normal weight, overweight and obese boys. LBM/height ratio showed that their low LBM is a result of true body composition difference rather than their shorter stature (Table 1). One of the factors for thinness in boys is due to malnutrition, but thin boys are not necessarily undernourished. The results of our longitudinal study confirm the findings that LBM had a major positive effect on bone mass parameters in boys as previously reported [9], [39]. We indeed found the positive effect of LBM on FN BMD and LS BMD parameters in pubertal boys with different body mass status, however for underweights the effect of LBM was not seen if controlling for the effects of SED time and VPA (see Table 4). This might be explained by an additional loading of the LBM, as it has been found in cross-sectional design that for overweight boys even moderate PA may be significantly related to bone mineral parameters [18].

In the current study to assess PA levels we used accelerometry, which could measure the amount, but not the type of PA. Although the association of activity duration has found to be greater compared to the frequency of the activity [40], there is also indication that the high strain eliciting or weight-bearing PA has a greater effect on bone mineral parameters, compared to the amount of PA [41]. At baseline only underweight and normal weight boys satisfied the recommendation of Physical Activity Guidelines for children and adolescents to spend at least 60 min in MVPA per day (see Table 1) [42]. Longitudinal multilevel model indicated that a boy with 60 min of MVPA had 0.00993 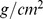 higher FN BMD compared to a boy of the same biological age, height and LBM, but with 30 min of MVPA a day (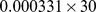; table 3). In comparison, after 24 months we had 35.4% of boys with 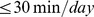 and only 5.4% of boys with 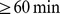 of MVPA in average (Table 2). In contrast, Gracia-Marco et al. [25] reported that more than 78 min/day of MVPA (>3 METs) or 32 min/day of VPA (>6 METs) was associated with increased FN BMD in adolescents, however, the study of Gracia-Marco et al [25] used epoch of 15 sec in accelerometry, while it was 60 sec in the current study, which might estimate differently, for example, the amount of vigorous PA. Our longitudinal analysis indicated that a boy with 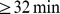 of VPA had 0.02273 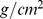 higher FN BMD compared to a boy of the same biological age, height and LBM, but with less than 10 min VPA a day 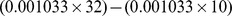
(Table 4). Furthermore, our data confirms the findings of Cardareiro et al. [43] who found that 10 min/day of VPA would be expected to result in a 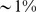 higher FN BMD in boys. Results from our study show 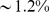 higher FN BMD in boys with every 10 min/day of VPA and 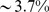 higher FN BMD in boys with 32 min/day of VPA.

Sedentary behavior is becoming more important and interesting topic in relation to bone mineralization, as it could result a higher bone resorption leading to reduced BMC (bone mineral content) [44]. Results from our study showed the significant increase in sedentary time over the 3-year period in all four groups of the boys (Table 2). Pate et al. (2008) reported that subject has to stay in low energy expenditure zone (<1.5 MET) for at least several hours to call it sedentary behavior [45]. The last year of the present study boys accumulated more than 9 hours of SED time (studying, watching television or surfing the internet) (Table 2). The study of Vicente-Rodriguez (2009) indicated that watching television for 3 or more hours a day is increasing risk for low BMC in male adolescents, however it is important to emphasize that this association is mediated by participation in PA and authors suggest that negative consequences of sedentary behavior on adolescent bone health could be counteracted by sport participation [46]. Similarly, Gracia-Marco (2012) found that use of the internet for non-study purposes was negatively associated with TB BMC and FN BMC in adolescent boys even after controlling for LBM and MVPA, also it was reported that total SED time was negatively associated with TB BMC in boys, but after controlling for LBM the association disappeared [47]. Our longitudinal models showed that SED time has a significant effect only on FN BMD (see Tables 3 and 4) and supports the main findings of the Gracia-Marco et al. (2012) study where they found negative association between total SED time and FN BMC after controlling for LBM and MVPA [47]. Our previous study regarding one year observation period indicated that SED time has an impact on bone mineral parameters, however using a longitudinal model the effect was stronger, which further indicates the need for longer observation periods in order to study the effect of sedentary time to bone mineral parameters [36]. Current and previously reported studies [36], [46], [47] provides important evidence of negative effect of SED time on bone mineralization in adolescents. However it is needed to carry out more longitudinal studies' to study the interaction between SED time and bone mass parameters.

Our presented models indicated that the group effect is not significantly important for FN BMD and LS BMD in boys, and that does not show differences between the groups when looking for effect of MVPA, VPA and SED time on FN BMD or LS BMD (see Tables 3 and 4). This indicates that being underweight, normal weight, overweight or obese boy at baseline (12 years old in our case) has no effect on future (2 years of follow up) FN BMD or LS BMD. However, there was a significant group effect of SED time, MVPA and VPA on TB BMD (P<0.001, see Tables 3 and 4). Further mixed procedure showed that being an underweight boy at baseline is related to higher chance to have lower TB BMD in the future (2 years at follow up) development compared to normal weight, overweight and obese boys (see Table 5). We have recently found that the cut-offs for PA, taking into account its effect on fitness and fatness parameters should be at least 59 min MVPA including 14 min VPA [43]. As the underweight boys fulfilled the criteria for healthy PA (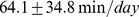), that even included 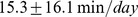 for VPA), it can be considered that the PA guidelines [25] for healthy bone development may not be sufficient for underweights. This result is very relevant, taking into account that femoral neck is very important for its clinical relevance to osteoporosis. Unfortunately, due to too small sample size in underweight, overweight and obese groups we could not run multilevel model for sufficient power for each group separately to check the effects of MVPA, VPA and SED time on TB BMD. However, there was a tendency that MVPA and VPA had a significant effect on TB BMD in overweight boys (data not shown). In underweight and normal weight boys, only LBM had a strong positive effect on TB BMD and in obese boys nor LBM, MVPA or SED had an effect on TB BMD (data not shown).

Our research has some limitations. Our major limitation is a relatively small number of subjects in underweight, overweight and obese groups. Because of that we could not run group-based longitudinal multilevel models for sufficient power. Further, the use of 1-min epoch in accelerometry might have some effect on short bouts of VPA and therefore, might probably underestimate the amount of VPA [48]. However, the strengths of the current study is the relatively long investigation period that covers two years during the puberty ending with PHV with objective measures of PA and bone mineral density.

## Conclusions

This study is unique in examining longitudinally the influence of PA and body composition on BMD parameters in boys during pubertal growth spurt. Our longitudinal multilevel models indicated that the LBM had a positive significant effect on TB BMD, FN BMD and LS BMD in boys during pubertal growth spurt. MVPA, VPA and SED time had significant effect only on FN BMD. Being an underweight boy at the baseline indicated greater chance (p<0.01) to have lower TB BMD in the future (2 years at follow up) development, compared to normal weight (estimates = −0.038), overweight (estimates = −0.061) and obese boys (estimates = −0.106).
